# On Efficacy of Microwave Ablation in the Thermal Treatment of an Early-Stage Hepatocellular Carcinoma

**DOI:** 10.3390/cancers13225784

**Published:** 2021-11-18

**Authors:** Branislav Radjenović, Martin Sabo, Lukaš Šoltes, Marta Prnova, Pavel Čičak, Marija Radmilović-Radjenović

**Affiliations:** 1 Institute of Physics, University of Belgrade, Pregrevica 118, 11080 Belgrade, Serbia; bradjeno@ipb.ac.rs; 2 Faculty of Informatics and Information Technologies, Slovak University of Technology in Bratislava, Ilkovicova 2, 84216 Bratislava, Slovakia; martin.sabo@stuba.sk (M.S.); lukas.soltes@stuba.sk (L.Š.); marta.prnova@stuba.sk (M.P.); pavel.cicak@stuba.sk (P.Č.)

**Keywords:** hepatocellular carcinoma, microwave ablation, necrotic tissue

## Abstract

**Simple Summary:**

Hepatocellular carcinoma accounts for around 75% of all liver cancers, and represents the fourth most common cause of cancer-related deaths worldwide. Microwave ablation is a worldwide-diffused treatment of hepatocellular carcinoma. According to the literature, the success rate for completely eliminating small liver tumors in patients treated with microwave ablation is greater than 85%. Microwave ablation is also highly recommended for COVID-19 patients with liver tumors as a fast treatment with a short recovery time. The involvement of the temperature dependence of the heat capacity, the thermal conductivity, and blood perfusion, is pivotal for establishing the correct ablation process and preserving the healthy tissue. The obtained simulation results clearly show that precisely localized heating distributions and heating efficiency can be achieved by using a multislot antenna probe. Deeper knowledge in this area would aid in the prediction and planning of patient-individual procedures.

**Abstract:**

Microwave ablation at 2.45 GHz is gaining popularity as an alternative therapy to hepatic resection with a higher overall survival rate than external beam radiation therapy and proton beam therapy. It also offers better long-term recurrence-free overall survival when compared with radiofrequency ablation. To improve the design and optimization of microwave ablation procedures, numerical models can provide crucial information. A three-dimensional model of the antenna and targeted tissue without homogeneity assumptions are the most realistic representation of the physical problem. Due to complexity and computational resources consumption, most of the existing numerical studies are based on using two-dimensional axisymmetric models to emulate actual three-dimensional cancers and surrounding tissue, which is often far from reality. The main goal of this study is to develop a fully three-dimensional model of a multislot microwave antenna immersed into liver tissue affected by early-stage hepatocellular carcinoma. The geometry of the tumor is taken from the 3D-IRCADb-01 liver tumors database. Simulations were performed involving the temperature dependence of the blood perfusion, dielectric and thermal properties of both healthy and tumoral liver tissues. The water content changes during the ablation process are also included. The optimal values of the input power and the ablation time are determined to ensure complete treatment of the tumor with minimal damage to the healthy tissue. It was found that a multislot antenna is designed to create predictable, large, spherical zones of the ablation that are not influenced by varying tissue environments. The obtained results may be useful for determining optimal conditions necessary for microwave ablation to be as effective as possible for treating early-stage hepatocellular carcinoma, with minimized invasiveness and collateral damages.

## 1. Introduction

Liver cancer is the abnormal growth of cells arising in the liver (primary) or spreading to the liver from primary cancer somewhere else in the body (secondary) [1,2,3,4,5,6,7,8]. The most prevalent form of primary liver cancer is hepatocellular carcinoma (HCC), which may start as a single tumor that grows or as a series of small cancer nodules forming throughout the liver [9,10,11,12,13,14]. Other types of liver cancer, such as intrahepatic cholangiocarcinoma, hepatoblastoma, angiosarcoma, and hemangiosarcoma are much less common [15,16,17,18]. Liver cancer is not only one of the most common cancers in the world, but also the fastest-growing cause of cancer death [19,20,21,22]. Thus, recognizing the safest and most efficient treatments for liver cancer has never been more urgent. Among various procedures, microwave ablation is an extremely promising, heat-based, minimally invasive thermal ablation modality in treating hepatic malignancies [23,24,25].

Although HCC is a highly aggressive cancer that accounts for more than 75% of all liver cancers, it has limited therapeutic options [26]. The most persuasive treatment modalities capable of achieving a cure are hepatic resection and hepatic transplantation. However, for patients that are not candidates for these therapies, the treatment option is highly individualized, depending on the type and stage of the HCC [27]. Patients in very-early (single lesion < 2 cm) and early-stage (single lesion < 5 cm or 2–3 nodules < 3 cm) HCC can be effectively treated with ablative therapies such as radiofrequency ablation (RFA) or microwave ablation (MWA) [28,29]. While RFA is recognized as a method with a very low rate of procedure-related morbidity and almost zero mortality [30], MWA provides some additional benefits [31]. Firstly, the treated tissue can be larger, while the treatment duration is shorter. Further, MWA is less affected by the defense of the neighboring tissues due to vaporization, and is less susceptible to the heatsink effect generated by the cooling effect of blood flow. Unlike RFA, MWA is not limited by tissue conductance since the propagation of energy does not depend on electrical tissue properties [31]. For HCC lesions smaller than 3 cm, both techniques are safe, with similar survival rates [32]. Transarterial chemoembolization (TACE) is the appropriate treatment of intermediate-stage HCC, while for advanced-stage disease, sorafenib is the only approved front-line molecular-targeted treatment [33,34,35]. Furthermore, a recent evaluation the of efficacy and safety of percutaneous MWA versus TACE, even for large HCC (5–7 cm), has shown that MWA displayed a lower incidence of tumor recurrence, de novo lesions, or post-treatment ascites [36].

MWA is a thermal ablation modality based on increasing the temperature above the normal physiological threshold to kill cancer cells with minimal damage to surrounding tissues [37,38,39,40,41]. During MWA a rapidly oscillating electromagnetic field leads to frictional heating of water molecules in the soft tissues around the field source [42,43]. The currents in the antenna, which are the source of the microwave fields, are also affected by the surrounding tissue impacting the antenna ablation performance and unpredictable ablation zones. One of the most sophisticated antenna designs achieves this by three different mechanisms: thermal control, field control, and wavelength control [44].

The initial MWA systems were constrained by poor antenna design and the inability to achieve spherical ablation zones, causing significant damage to adjacent healthy tissues [45,46]. The major limitation of earlier MWA devices is the lack of predictability of the ablation zone size and shape, since it depends on the inherent characteristics of the target tissues. Various antennas for ablation of near-spherical tumors, including choke, cap-choke, floating sleeve, and water-cooled antennas have been developed [47,48,49,50]. Some modern, commercially available MWA systems rely on the, so-called, “thermosphere technology”, that enables the formation of spherical ablation zones by implementing small saline irrigation channels in the antenna, thus making them independent of tissue properties [51,52]. Recently, a compact, multislot coaxial antenna was built to obtain the required ablation shape and proper impedance matching to the target tumor tissue without damaging the surrounding healthy tissues [53,54], so this design shall be used in the present study.

In further developments and improvements of the design and optimization of microwave ablation devices, a numerical model of the antenna-tissue system play a central role in providing vital information on the thermal behavior of the tissue [55,56,57,58,59]. The performance of the antenna has been estimated considering the specific absorption rate, antenna impedance, and geometry of the obtained thermal lesion [60,61,62,63,64]. Several numerical works are devoted to the prediction of the temperature profile in the tissue and resultant tissue damage created by ablation devices [65,66]. In most of the studies, the Arrhenius model is used to estimate the degree of destruction of biological tissue [67]. For studying the influence of the shape and size of the tumor during MWA, coupled bioheat and electromagnetic equations are solved by using the finite element method (FEM) [68]. Most of the numerical studies related to MWA refer to two-dimensional (2D) axis-symmetric simulations assuming a homogeneous medium [69]. This reduces the problem from a three-dimensional (3D) to a 2D one, which is often far from reality.

In this paper, we developed and tested a full 3D model of the microwave ablation process with realistic geometry of the liver tumor taken from 3D-IRCADb-01 liver tumors database [70]. The Comsol Multiphysics simulation package has been used as a platform to solve the coupled electromagnetic–thermal problem of MWA [71,72]. As a source of microwaves, we shall use a compact 10-slot coaxial antenna with a pi impedance-matching network that creates near-spherical ablation zones [53,54]. The finely tuned pi impedance matching eliminates the damaging of the surrounding healthy tissues. Simulations were performed for an antenna operating at 2.45 GHz inserted into the tumor, including the temperature dependence of dielectric and thermal properties of healthy and malignant liver tissues, the blood perfusion, and water content. The power dissipation, the time evolution of the temperature, and the degree of tissue destruction under the influence of high temperature have been estimated. The ultimate goal of the developed simulation model is to help practitioners to determine optimal input power of the ablation device and duration of the ablation process for the actual shape of the patient tumor and chosen position of the ablation probe.

## 2. Numerical Method and Simulation Conditions

Every mathematical model for the simulation of microwave ablation consists of three fundamental components. The first component is the model of the antenna probe (or applicator) that generates a microwave field in the tissue. The second component describes the heat distribution in the tissue including sources and sinks and the phase changes. In our case, the microwave field is the source of heat, and the heat sinks are represented by the blood perfusion term in the heat transfer equation. The third part deals with the effect of heat on tumor cells and their destruction. All these components of the ablation model depend on a variety of material parameters, which themselves depend on the various states of the tissue.

**Figure 1:**
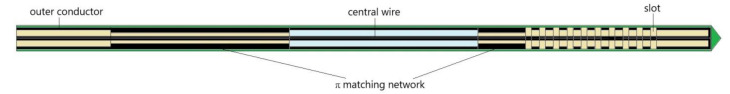
Schematic view of the 10-slot microwave antenna with an impedance match network. Black, green, light blue, and brown colors correspond to conducting material, Teflon, air, and dielectric, respectively. The width of the slot is 0.6 mm, while the spacing between slots is 0.8 mm.

For small tumors or tumors adjacent to vital organs, MWA with antennas operating at 2.45 GHz is recommended due to its more localized ablation zone [26,73]. The antennas are usually mechanically and geometrically complex, and the simulation relies on having accurate electromagnetic material and tissue properties. In this study, we use a compact 10-slot microwave antenna with an impedance pi-matching network that creates near-spherical ablation zones, schematically represented in Figure 1 [53,54]. The finely tuned, impedance matching eliminates the damaging of the surrounding healthy tissues. The multislot radiating probe is composed of several periodic elements equal to a linear uniform antenna array. Each periodic element individually comprises a slot with a width of 0.6 mm and a spacing conductor of 0.8 mm between adjacent slots. The required ablation shape is achieved by optimizing the distance between the adjacent slots and the number of the slot. In comparison with previously developed antennas [74,75,76,77], this multislot coaxial antenna produces a more localized heating pattern and a smaller overtreatment region for spherical liver tumors.

**Figure 2:**
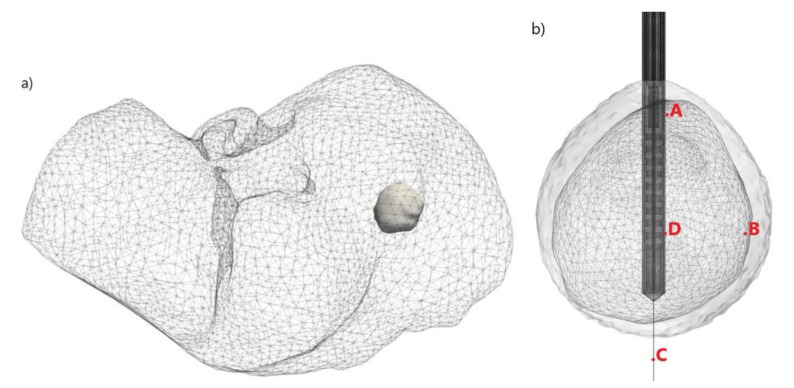
Three-dimensional (**a**) simulation model of the liver (triangulated surface) and an early-stage HCC (solid surface) corresponding to patient 16 in the 3D-IRCADb-01 database [70] and (**b**) configuration of the antenna inserted into biological tissue and positions of four test points (A, B, C, D).

To define as realistic a simulation model as possible, we did not use a spherical tumor geometry, as is usually the case. In our analysis, we shall use the data from the 3D-IRCADb-01 database [70] that includes several sets of CT scans of patients manually segmented by clinical experts. We perform a simulation on the data reported for patient 16 in the database. The shapes of the tumor (and the surrounding liver) are shown in Figure 2a. Figure 2b illustrates a 10-slot microwave antenna inserted into a liver tumor with four test points (A, B, C, and D) marked in red, blue, green and purple, and located at different sites. Points A and D are positioned close to the heating center along the antenna shaft. Points B and C were placed along with the radial direction corresponding to the maximum transverse of each antenna. At these points, we shall follow the time dependence of temperature and tissue damage during the ablation process. They correspond to the usual positions of thermocouples in experimental studies [54].

For this study, the 3D finite elements method (FEM) is used to solve coupled electromagnetic-field and heat-transfer equations, including all details of antenna design and properties of healthy and tumoral tissue. Our 3D model is created within the COMSOL Multiphysics FEM-based simulation platform [68,71]. Equations that describe the propagation of microwaves in tissue by an antenna have forms [71,72]:

(1) $$\nabla^{2}E-\mu_{r}k_{0}^{2}\left(\varepsilon_{r}-\frac{j\sigma }{\omega \varepsilon_{0}}\right)E=0,$$

with the angular frequency *ω*, the vacuum propagation constant *k*_0_ = *ω*/*c*_0_, the electric field vector ***E***, and the tissue electrical conductivity *σ.* *ε*_0_, ε*_r_,* and *μ_r_* are the vacuum dielectric constant, relative permittivity, and permeability of the tissue, respectively.

**Figure 3:**
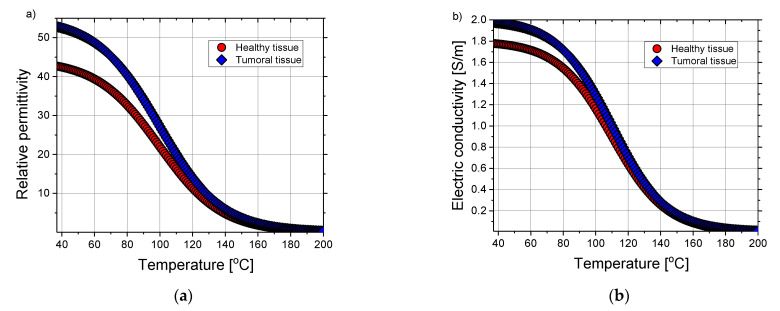
The temperature dependence of (**a**) relative permittivity and (**b**) electric conductivity of the healthy (red circles) and tumoral liver tissue (blue diamonds). Plots are obtained by using expressions (2) and (3) with coefficients taken from the literature [68].

During MWA, the elevated temperatures reached by the tissue close to the antenna cause structural modifications of treated tissue, resulting in changes in the dielectric and thermal properties that affect the electromagnetic power distribution. Since dielectric properties of the tissue are temperature-dependent [78,79], we used the expressions [68,80]:

(2) $$\varepsilon_{r}\left(T\right)=s_{1}\left[1-\frac{1}{1+\exp\left(s_{2}-s_{3}T\right)}\right],$$

(3) $$\sigma \left(T\right)=r_{1}\left[1-\frac{1}{1+\exp\left(r_{2}-r_{3}T\right)}\right],$$

with coefficients taken from [68]. Figure 3 displays the sigmoidal temperature-dependent model of (a) relative permittivity and (b) electric conductivity for healthy and tumoral tissues. As expected, relative permittivity and conductivity of liver tumors are around 24% and 11% higher than those corresponding to healthy liver tissue [81]. Due to the water evaporation during the MWA, dielectric properties of tissue decrease with increasing the temperature [82]. Additionally, the change rate of dielectric properties with temperature is the same in both healthy and tumoral tissues.

Heat transfer during the MWA process can be accurately described by the Pennes bioheat equation [83]:

(4) $$\rho c\frac{\partial T}{\partial t}=\nabla \times \left(k\nabla T\right)+\rho_{b}W_{b}c_{b}\left(T_{b}-T\right)+Q_{ext}+Q_{m},$$

where *t* is time, *ρ*, *c*, and *T* are the density, the heat capacity, and the temperature of the tissue, respectively, and *ρ*_b_, *c*_b,_  *T*_b,_ and *W*_b_ are the density, the heat capacity, the temperature, and the perfusion rate of the blood, respectively. The heat source from metabolism *Q*_m_ is neglected in our calculation, while the external heat source *Q*_ext_ describes coupling with electromagnetic field and is given by:

(5) $$Q_{\mathrm{ext}}=\frac{\sigma {\left|E\right|}^{2}}{2}.$$

The tissue thermal conductivity *k* varies with the temperature [68,84]:

(6) $$k\left(T\right)=k_{0}+\Delta k\left|T-T_{0}\right|,$$

where *k_0_*  *is* the thermal conductivity measured at temperature *T_0_*, while Δ*k* represents the change in *k* due to temperature. Blood perfusion dictates the bioheat transfer in living tissues. The difference in temperature between blood and tissue leads to convective heat transfer, so the blood perfusion rate *ω*_b_ is also a function of temperature [68]:

(7) $$\omega_{b}=2.1\times 10^{-5}T+3.5\times 10^{-3}.$$

Another important temperature-dependent parameter is water vaporization, which influences specific heat. The water content *W* is given by the expression [85]:

(8) $$\begin{array}{l} W(T)=\left\{ \begin{array}{l} 0.778\cdot \left(1-e^{\frac{T-106}{3,42}}\right),\;\;70\;{}^{\circ}C\leq T<100\;{}^{\circ}C\\ 7.053-0.064096\cdot T,\;\;100\;{}^{\circ}C\leq T<104\;{}^{\circ}C\\ 0.778\cdot e^{-\frac{T-80}{34.37}},\;\;T\geq 104\;{}^{\circ}C \end{array}\right. \end{array}$$

**Figure 4:**
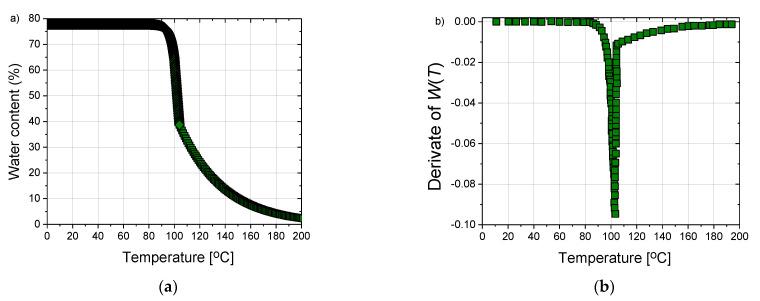
(**a**) The time dependence of the water content *W*(*T*) of the tissue according to expression (8), taken from reference [85] and (**b**) the first derivate of *W*(*T*) which is then used to calculate an effective specific heat given by Equation (9).

At steady state, the water content of liver tissue is around 78% water by mass, as depicted in Figure 4a. For temperatures above 100 °C, the tissue water content may decrease to less than 20% by a mass due to evaporation, leading to drastic changes in tissue dielectric parameters and greater penetration of microwaves [82,85]. Since a large fraction of liver tissue consists of water, the tissue’s thermal properties are similar to those of water and vary with temperature and water content [86]. It is shown in [87] that the influence of internal water evaporation can be included in bioheat Equation (4) by replacing specific heat *c* with an effective value *c’*, given by the relation:

(9) $$c^{’}=c-\frac{\alpha }{\rho }\frac{\partial W}{\partial T}$$

where *α* is the water latent heat constant equal to 2260 (kJ/kg) [85]. The derivative of the *W*(*T*) appearing in the above relation is shown in Figure 4b.

The biological damage depends on both temperature and time. Although tissue damage can be associated with many different reactions, it may be approximated in a single process characterized by a single rate constant of the Arrhenius form [88]. An arbitrary function of tissue injury *Ω* is defined as [89]:

(10) $$\Omega \left(t\right)=\int_{0}^{t}A\exp\left(-\frac{\Delta E}{RT}\right)dt,$$

where *A* and Δ*E* represent the frequency factor and the activation energy for irreversible damage reaction, respectively, *T* is the temperature calculated at each point of the model region and *R* is the gas constant. Fraction of necrotic tissue, *θ*_d_, can be determined from the degree of tissue injury *Ω* [65]:

(11) $$\theta_{d}=1-\exp\left(-\Omega \right).$$

Numerical simulations were performed for the microwave frequency of 2.45 GHz and the input power of 13 W, including the temperature dependence of the dielectric properties of tissues, thermal conductivity, heat capacity, and blood perfusion. Parameters that characterize healthy liver, tumoral tissue, and blood are found in the literature [68] and listed in Table 1.

## 3. Results

**Figure 5:**
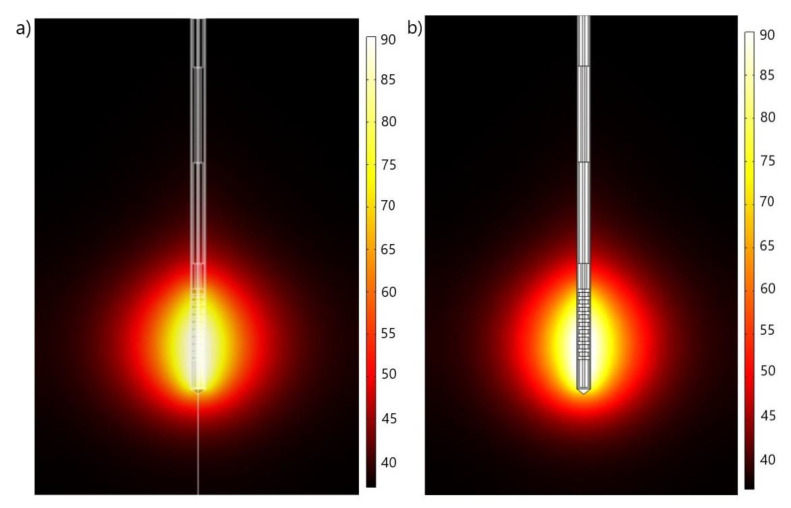
Results of (**a**) two-dimensional axial-symmetric and (**b**) full three-dimensional simulation models of the temperature (expressed in °C) distributions in the liver tissue after 600 s of microwave ablation at a frequency 2.45 GHz and the input power of 10 W.

Figure 5 contains the results of the test calculations for the liver tissue exposed to microwave frequency of 2.45 GHz and the input power of 10 W by using (a) 2D axial-symmetric and (b) full 3D simulation models. As can be observed, the temperature distributions obtained by both models are in an excellent agreement, confirming that the full 3D model that we developed could provide correct results and could be useful for realistic modelling of the effect of MWA on early-stage HCC.

**Figure 6:**
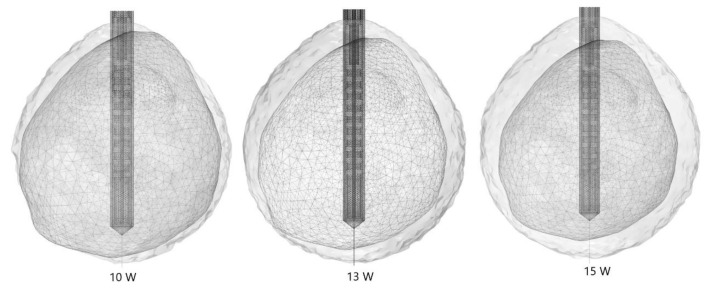
Isocontours represent the totally ablated regions (gray surfaces) around the liver tumor [70] (triangulated surface) exposed to 600 s of microwave ablation at 2.45 GHz and the three values of the input power (10 W, 13 W, and 15 W).

To determine the proper value of the input power that leads to the minimal tissue damage, calculations were carried out for a frequency of 2.45 GHz, ablation time of 600 s, and three values of the input power (10 W, 13 W, and 15 W). The optimal input power that causes minimal healthy tissue damage can be estimated from the isocontours related to the fraction of damage equal to 1, shown in Figure 6. For the input power of 10 W, tumoral tissue is not treated completely with MWA. When the input power is 15 W, the ablation zone encompasses the whole tumor, but some parts of healthy tissue are also damaged. The isocontour that best fits the necrotic tissue corresponds to the input power of 13 W, where the tumor is totally treated with minimal damage to the healthy tissue. Since the proper choice of the input power strongly depends on the size and the shape of the tumor, they have to be determined before any procedure in order to achieve the desired ablation margin.

**Figure 7:**
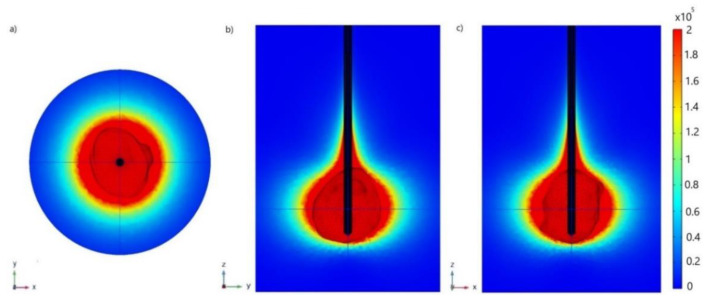
(**a**) *x–y*, (**b**) *y–z*, and (**c**) *x–z* cut planes of the total power dissipation density (expressed in W/m^3^) calculated for the liver tumor [70] (plotted as triangulated surface) exposed to microwave frequency of 2.45 GHz and input power of 13 W after 600 s.

Figure 7 shows (a) *x–y*, (b) *y–z*, and (c) *x–z* cut planes of the microwave power density absorbed in tumoral liver tissue during MWA at 2.45 GHz and input power of 13 W at the end of the 600 s ablation process. A microwave field oscillates rapidly, causing rotation of polar molecules, primarily water, and some amount of electromagnetic energy is absorbed. Close to the antenna, absorbed power density is large, and this decreases with distance. The energy emitted from the antenna through the tissue is converted into heat that destroys cancer cells. The majority of the heat is due to the excitement of polar water particles, while ionic polarization contributes to a minor part of the generated heat. The heated zone is almost spherical and encompasses the tumoral tissue. The input power and ablation time are chosen so that a very small area of healthy tissue around the tumor is heated.

**Figure 8:**
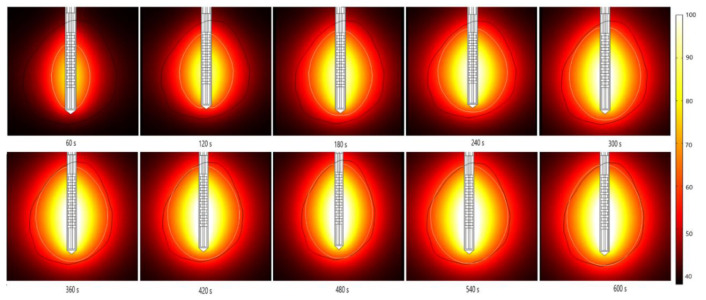
Temporal evolution of the temperature (in °C) when an early-stage HCC [70] is treated by microwave ablation at a frequency of 2.45 GHz and input power of 13 W in *y–z* cut plane. The boundary of the tumor tissue is marked by the black line.

The absorbed energy converted into thermal energy leads to an increase in the tissue temperature, as shown in Figure 8. The boundary of the tumoral tissue is designated by the black line, while the white line represents the 60 °C isothermal contours. It was reported that cell death occurs instantly above 60 °C [54], so the 60 °C isothermal contour is linked to the lesion size and shape of the ablated tissue. The perfusion of blood restricts the extent of the heated area. The temperature also rises with the ablation time, reaching a value of around 104 °C after 600 s. As expected, a multislot antenna structure enables more localized near-spherical heating distributions.

**Figure 9:**
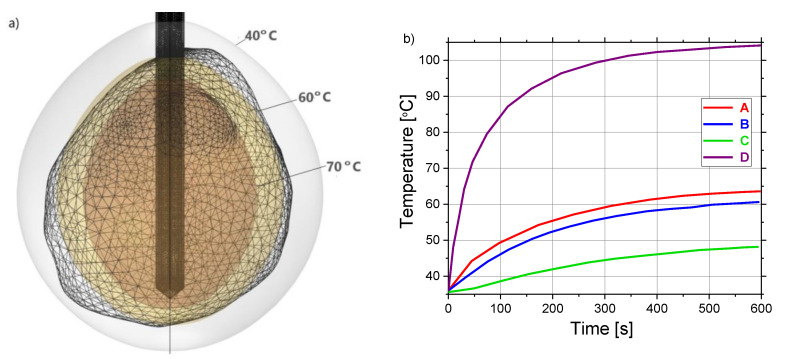
(**a**) Three-dimensional plot of the liver tumor [70] represented by triangulated surface and isocontours that correspond to the temperature of 40 °C (light gray), 60 °C (light brown), and 70 °C (brown). (**b**) The time dependence of the temperature calculated at test points (A, B, C, and D) is marked in Figure 4b.

Isocontours that correspond to the temperatures of 40 °C (light gray), 60 °C (light brown), and 70 °C (brown) around the tumoral tissue (triangulated surface) are plotted in Figure 9a. Close to the antenna the heat source is stronger, so the temperature is higher. The time dependence of the temperatures at four test points (A, B, C, and D) marked in Figure 4b can be seen in Figure 9b. All curves have the same tendencies for all test points. For channel D (located near the heating center), the temperature rapidly increased to approximately 79 °C after 70 s and then steeply rises up to 105 °C after 600 s, which is in line with the temperature distributions shown in Figure 8 and the data found in the literature [54]. In contrast, the lowest value of the temperature of around 48 °C after 600 s is calculated for channel C which is the most distant from the antenna. The maximal temperatures recorded at the points located along the radial direction concerning the maximum transverse of each antenna D (close to the heating center) and B (away from the heating center) differ by around 42%. On the other hand, the maximal value of the temperature at point D is larger by around 37% and 53% than those in points A and C distributed at a distance from the heating center along the antenna shaft, respectively.

**Figure 10:**
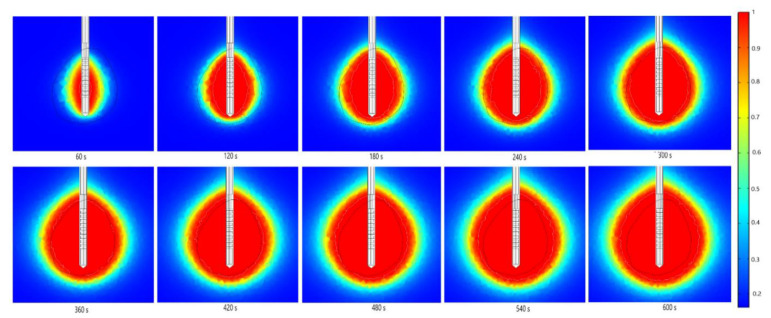
The time evolution of the fraction of necrotic tissue exposed to the microwave ablation at a frequency of 2.45 GHz and the input power of 13 W. The boundary of the tumoral tissue is marked by the black line.

Numerical predictions of the fraction of necrotic tissue during MWA at   a frequency of 2.45 GHz and the input power of 13 W are presented in Figure 10. The black line denotes the boundary of the tumoral tissue. Regardless of the time, the damage zones are concentrated around the tip and slots of the antenna, while the backward heating effect is smaller. The use of multiple slots in the antennas provided a more spherical ablation volume for the tissue heated. Thermally ablative devices lead to necrotic tissue with two distinct heating zones, recognized as an active heating zone and a passive heating zone [90]. The active heating zone arises within the tissue nearest to the device where the intensity of energy is high and its absorption by tissue is fast. On the other hand, the passive zone is outside the active zone, far from the ablation device where the intensity of energy is lower.

**Figure 11:**
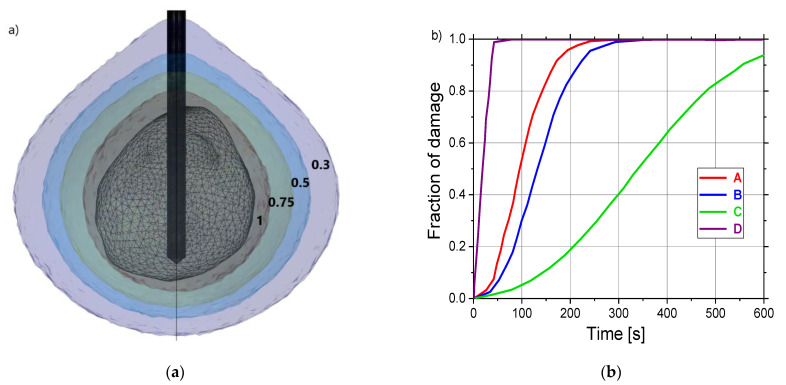
(**a**) The liver tumor [70] is shown as triangulated surface and isocontours corresponding to the fractions of damage of 0.3 (violet), 0.5 (blue), 0.75 (dark green), and 1 (dark gray). (**b**) The time evolution of the necrotic tissue was calculated at four test points (A, B, C, and D) marked in Figure 4b.

Isocontours related to fractions of damage of 0.3 (violet), 0.5 (blue), 0.75 (dark green), and 1 (dark gray) are shown in Figure 11a. Figure 11b demonstrates the time evolution of the necrotic tissue at four test points (A, B, C, and D) marked in Figure 4b. For test points A, B, and D, at the beginning of the ablation, the tumor damage gradually increases and then reaches a saturation region, which presents the completion time of tumor necrosis. The fastest completion of tumor necrosis is obtained for point D, while for point C the tumor necrosis is uncomplete.

## 4. Conclusions

This paper reports on simulation studies of the effect of microwave ablation on the early-stage hepatocellular carcinoma. For this purpose, a full three-dimensional model has been developed and tested within COMSOL Multiphysics, a finite element method-based platform [71]. Calculations were performed for a model of real liver tumor extracted from the database 3D-ICRADb-01 [70], exposed to radiation by a 10-slot microwave antenna operating at 2.45 GHz, combined with biological materials data collected from the literature [68]. The obtained simulation results show that full 3D simulation of the ablation process provides the best operating parameters (input power of 13 W and ablation time of 600 s) to achieve the greatest performance of MWA for this concrete, real, complex antenna and realistic tumor tissue [70]. Other antennas or tumor shapes will require different operating parameters. The inclusion of the temperature dependence of the heat capacity, the thermal conductivity, and blood perfusion, is important for calculating the correct ablation time, thus contributing to the preservation of the healthy tissue. One of the main characteristics associated with the efficiency of MWA is that temperature profile is mainly governed by the heat-source distribution. The temperature increases during ablation and reaches the maximal value near the microwave antenna slots. After achieving saturation, the diffusion and the heat conduction due to the blood perfusion become significant. Temperature distributions are almost spherical, and the damage zones are concentrated around the tip and slots of the antenna. The extension of the necrotic tissue increases, taking place mainly in the tumor, and only a small amount of surrounding tissue is damaged.

## Figures and Tables

**Table 1:** The parameters of biological materials (healthy liver tissue, tumoral liver tissue, and blood) were collected from the literature [68] and used in numerical simulations.

Parameter	Value
**Tissue properties**	
Density	1079 kg/m3
Thermal conductivity	0.52 W/m °C
Specific heat	3540 J/kg °C
**Tumor properties**	
Density	1040 kg/m3
Thermal conductivity	0.57 W/m °C
Specific heat	3960 J/kg °C
**Blood properties**	
Density	1060 kg/m3
Thermal conductivity	0.5 W/m °C
Specific heat	3600 J/kg °C
Temperature	37 °C

## Data Availability

The data are available from the corresponding authors upon reasonable request.
